# Invasive Apocrine Carcinoma in a Young Female With Triple Hormone Receptor Positivity: A Case Report

**DOI:** 10.7759/cureus.103977

**Published:** 2026-02-20

**Authors:** Umang K Agrawal, Sakshi Jaiswal

**Affiliations:** 1 General Surgery, Employees' State Insurance Corporation (ESIC) Medical College and Hospital, Varanasi, Varanasi, IND

**Keywords:** androgen receptor, apocrine breast carcinoma, breast conservation surgery, immunohistochemistry, triple hormone receptor-positive

## Abstract

Apocrine carcinoma (AC) of the breast is a rare histological subtype, classically characterized by androgen receptor (AR) positivity with estrogen receptor (ER) and progesterone receptor (PR) negativity. We report the case of a 35-year-old premenopausal woman who presented with a five-month history of a left retroareolar breast lump associated with intermittent serous nipple discharge. Clinical examination and breast imaging revealed a suspicious lesion, and core needle biopsy suggested invasive ductal carcinoma. The patient underwent nipple-sparing breast conservation surgery with axillary clearance. Histopathological examination demonstrated invasive AC, modified Bloom-Richardson grade 1, without lymphovascular invasion or nodal metastasis. Immunohistochemistry revealed an unusual triple hormone receptor-positive profile (ER-positive, PR-positive, AR-positive), with human epidermal growth factor receptor 2 (HER2) negativity and a low Ki-67 proliferative index. Adjuvant treatment included combination chemotherapy, radiotherapy, and dual hormonal therapy with tamoxifen and an AR inhibitor. This case highlights the diagnostic and therapeutic challenges of hormone receptor-positive AC and underscores the importance of comprehensive immunohistochemical profiling for individualized management.

## Introduction

Apocrine carcinoma (AC) accounts for 0.5-4% of invasive breast cancers [1,2] and is defined by cytological features resembling apocrine sweat glands, including abundant eosinophilic cytoplasm and prominent nucleoli [3]. Historically, AC is classified as ER/PR-negative and AR-positive, often falling into the triple-negative or HER2-positive categories [4,5]. However, rare cases exhibit ER/PR positivity, complicating diagnostic and therapeutic paradigms [6]. The World Health Organization (WHO) mandates >90% apocrine morphology for a "pure" AC diagnosis, supported by GCDFP-15 immunohistochemistry (IHC) [7]. Prognosis remains debated, with some studies suggesting outcomes comparable to invasive ductal carcinoma (IDC), while others associate AC with poorer survival [7,8]. Emerging evidence highlights AR’s role as a therapeutic target, particularly in ER/PR-negative cases, although data on triple hormone receptor-positive AC are rare.

This report details a young patient with ER/PR/AR-positive AC, emphasizing diagnostic challenges, therapeutic strategies, and literature review.

## Case presentation

**Figure 1:**
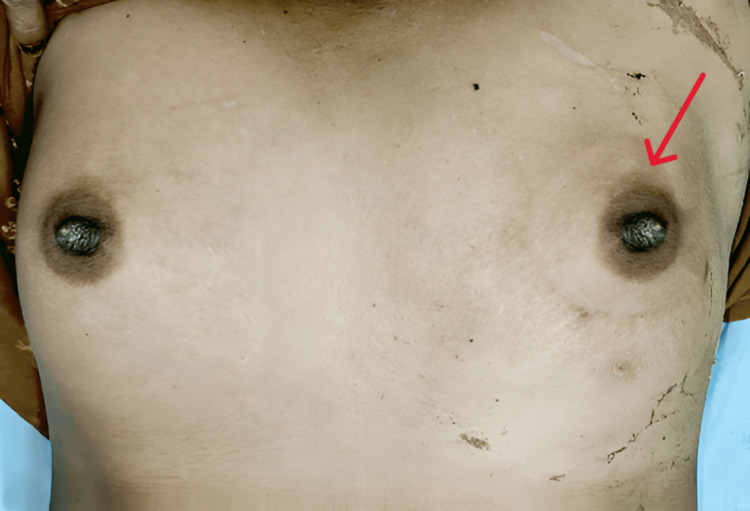
Clinical picture at presentation with the left breast lump

A 35-year-old premenopausal female, with no family history of breast cancer, presented at Employees’ State Insurance Corporation (ESIC) Hospital, Varanasi, with a five-month history of a left breast lump and intermittent serous nipple discharge. Clinical examination revealed a 3 × 4 cm, well-defined, firm, and mobile lump in the retroareolar region of the left breast (Figure 1). 

No axillary or supraclavicular lymphadenopathy was noted. The clinical staging of the patient was cT2N0M0. Ultrasonography of the bilateral breast with axilla showed a heterogeneous, hypoechoic mass (3.2 × 4.1 cm) with irregular margins and posterior acoustic shadowing, with a 1 x 1 cm axillary lymph node categorized as BI-RADS 2 lesion in the left breast. The right breast showed features suggestive of fibroadenosis.

**Figure 2:**
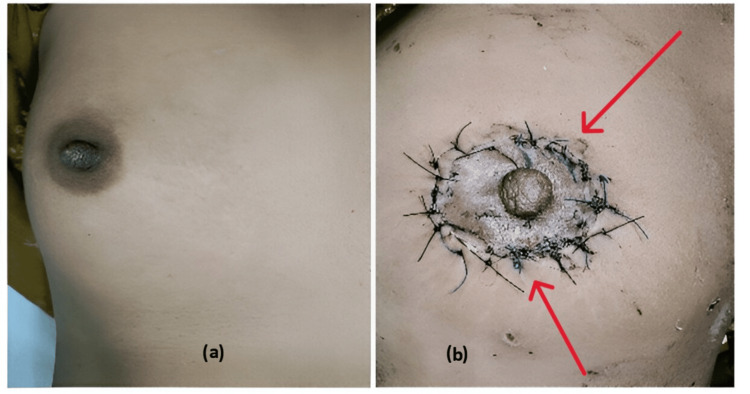
(a) Clinical picture of the normal right breast. (b) Clinical picture following lumpectomy and donut mastopexy of the left breast.

The core needle biopsy from the left breast lump showed the features of invasive ductal carcinoma. The patient underwent a nipple sparing surgery; donut mastopexy of the left breast, along with axillary clearance (Figure 2b). The breast lump specimen was sent to the department of pathology for histopathological examination (HPE) and Immunohistochemistry.

HPE showed tumor cells forming crowded tubules, small solid nests, and papillae having cytological features of apocrine cells with clear margins and no lymphovascular invasion, with the rest of the breast tissue showing features of fibrocystic disease with no nodal metastasis (modified Bloom Richardson Grade 1 score 4) [9].

**Figure 3:**
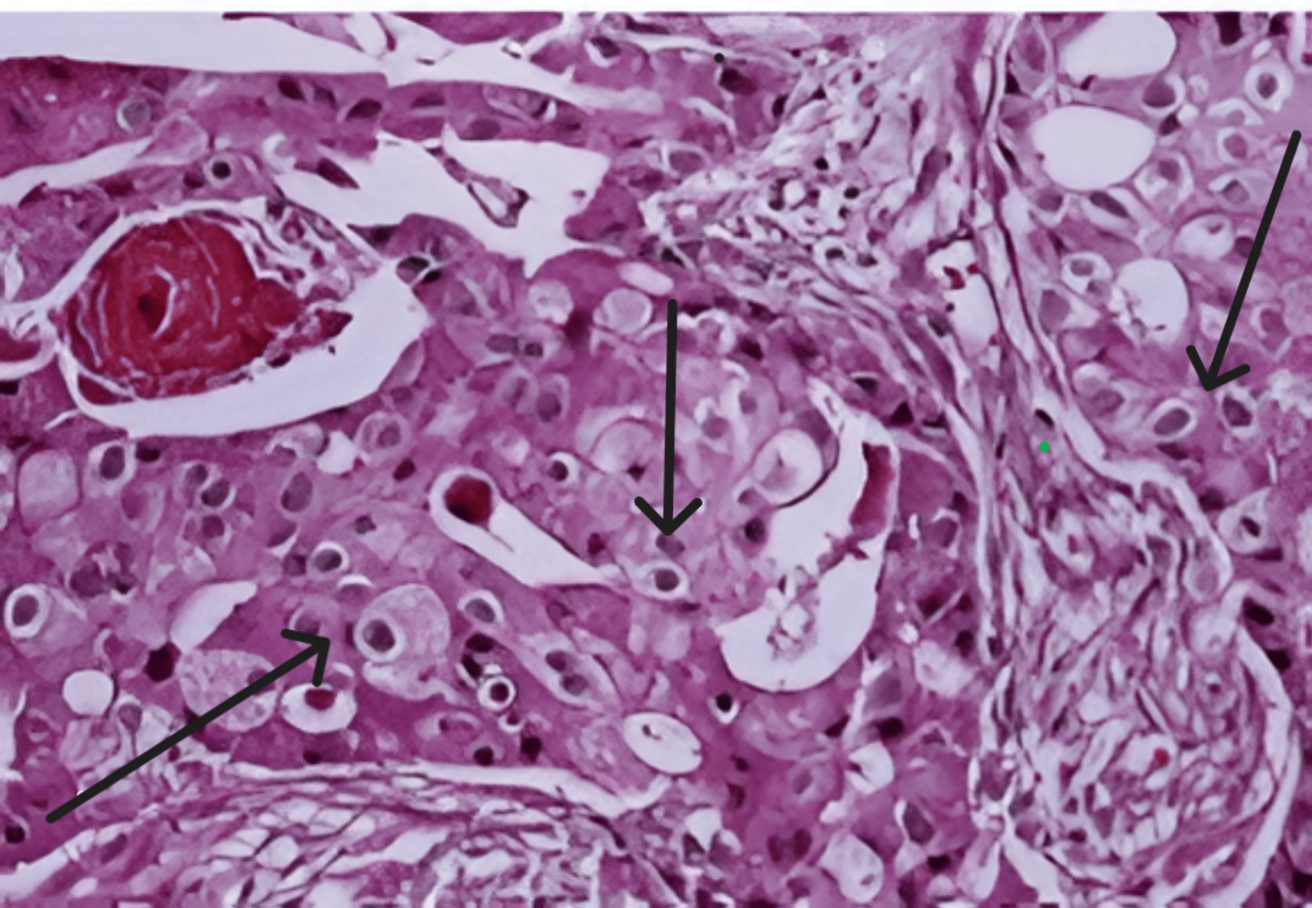
Apocrine cells: polygonal cells with granular eosinophilic cytoplasm, vesicular nuclei, and prominent nucleoli

Immunohistochemistry (IHC) profile showed ER of 50% strong nuclear positivity, PR of 20% moderate nuclear positivity, AR of 90% strong nuclear positivity, HER2 of 0 (negative), and Ki-67 of 2% with apocrine features, i.e., polygonal cells with granular eosinophilic cytoplasm, vesicular nuclei, and prominent nucleoli (Figure 3).

The patient was planned for adjuvant chemotherapy involving three rounds of Adriamycin, cyclophosphamide, and 5-fluorouracil (CAF), followed by three cycles of taxanes (paclitaxel). Twenty-one days after the last administration of CAF chemotherapy, paclitaxel was initiated and given every 21 days [10,11,12]. Four weeks after completing the last cycle of paclitaxel, the patient was given adjuvant radiotherapy, specifically targeting the supraclavicular zone and left breast [13]. Tamoxifen (20 mg/day) and the androgen receptor (AR) inhibitor, bicalutamide (50 mg/day) was planned for five years post-adjuvant chemotherapy. The patient is being followed up at regular intervals in the General Surgery OPD at ESIC Hospital, Varanasi.

## Discussion

This case of triple hormone receptor-positive: ER/PR/AR positive, invasive apocrine carcinoma (AC) in a young female presents several clinically and biologically significant nuances that challenge conventional paradigms. Below, we explore these aspects in depth, contextualizing them within existing literature and highlighting implications for diagnosis, therapy, and prognosis.

AC is traditionally defined by ER/PR negativity and AR positivity, often categorized as triple-negative (TNBC) or HER2-positive [5]. However, this case exhibited ER (50%) and PR (20%) expression alongside strong AR positivity (90%), a profile rarely documented in AC [6]. Dellapasqua et al. (2013) [6] reported that only 10-30% of AC cases demonstrate hormone receptor expression, often at low levels, suggesting that ER/PR positivity may represent a distinct luminal-apocrine hybrid phenotype. This aligns with Vranic et al. (2015) [7], who proposed molecular subtyping of AC based on AR and HER2 status, but our case further complicates this framework by exhibiting luminal B-like features (ER/PR+, HER2−, low Ki-67).

The WHO criteria for "pure" AC require >90% apocrine morphology and GCDFP-15 positivity [6,12]. While our case met morphological criteria, the ER/PR expression raises questions about whether such tumors should be reclassified or recognized as a unique subtype. This diagnostic ambiguity underscores the need for updated guidelines integrating immunohistochemical and molecular profiling to better stratify AC variants.

Therapeutic decisions for AC are often extrapolated from TNBC or luminal carcinoma protocols due to limited evidence. In this patient, the combination of tamoxifen (anti-estrogen) and bicalutamide (AR inhibitor) was selected to target both ER and AR pathways, a strategy supported by preclinical models. Cochrane et al. (2014) [5] demonstrated that AR blockade synergizes with endocrine therapy in ER+/AR+ breast cancer models, suppressing tumor growth more effectively than either agent alone. Mills et al. (2016) [14] further validated this approach in a cohort of AR-positive AC patients, showing prolonged survival with dual hormonal therapy.

The choice of the CAF regimen (cyclophosphamide, anthracycline, and fluorouracil) was guided by the patient’s young age and the moderate proliferative index (Ki-67: 2%). While young age often correlates with aggressive biology, the low Ki-67 and absence of lymphovascular invasion suggested indolent behaviour, prompting a balanced approach to avoid overtreatment. Post-surgical radiotherapy followed hypofractionated protocols (50 Gy/25 fractions + boost) as per Wang et al. (2019) [10], which demonstrated equivalent efficacy and reduced toxicity compared to conventional fractionation in high-risk breast cancer. Patients with AC are typically diagnosed in older women (median age: 60-70 years) [14,15], making this 35-year-old patient an outlier. While Mills et al. (2016) [14] associated younger age with favorable outcomes in AR-positive AC, larger studies are lacking. Zhang et al. (2017) [16] reported comparable survival between AC and invasive ductal carcinoma (IDC), but their analysis did not stratify by age or hormone receptor status. In our case, the absence of nodal metastasis (pT2N0M0), low Ki-67, and triple hormone positivity may collectively portend a better prognosis. However, the long-term impact of AR inhibition in ER+/PR+ AC remains unknown, necessitating vigilant follow-up [17]. The rarity of AC has hindered large-scale studies, leaving critical gaps in knowledge.

## Conclusions

This case illuminates the diagnostic and therapeutic complexities of triple hormone receptor-positive AC, challenging the traditional view of AC as a solely ER/PR-negative entity. The integration of AR blockade with endocrine therapy represents a novel strategy that may improve outcomes in hormone-responsive subsets. However, the field needs multicenter collaborations to establish standardized diagnostic criteria and evidence-based treatment protocols. As precision oncology advances, molecular subtyping and targeted therapies will likely redefine the management of this rare malignancy.
